# “Speak Up!” Investigating U.S. professional sports teams' #BlackLivesMatter statements

**DOI:** 10.3389/fspor.2023.1192784

**Published:** 2023-05-26

**Authors:** Dae Hee Kwak, Sean Pradhan, Zhjing Chen

**Affiliations:** ^1^ Department of Sport Management, University of Michigan, Ann Arbor, MI, United States; ^2^ Department of Management, Menlo College, Atherton, CA, United States

**Keywords:** text analysis, sentiment analaysis, social activism, public relations, professional sports

## Abstract

George Floyd's death caused by police brutality fueled a wave of the Black Lives Matter (BLM) movement both nationally and globally. Almost every professional sports team in the United States released a statement pertaining to racial inequality and social injustice. The current study investigated the content and word counts of the BLM statements posted on Twitter by all teams across the four major men's professional sports leagues: Major League Baseball (MLB), National Basketball Association (NBA), National Football League (NFL), and National Hockey League (NHL). Based on a series of text analyses, we found differences in both the content and word counts of statements put forth by each league. Notably, compared to teams in other leagues, NFL teams avoided negative sentiment words (e.g., by not using words like “racism”) and utilized more action-oriented terms like “support”, “listen”, and “conversation” in their statements. Practical implications and future directions for research are discussed.

## Introduction

Sports have often been considered a viable and effective platform to raise awareness and promote conversations around important social issues. Although the call for anti-racism in sports is not new (e.g., 1), the intensity of its call gained a new momentum in the wake of the Black Lives Matter (BLM) movement (e.g., 2–4). In particular, the murder of George Floyd caused by police brutality in May 2020 intensified the calls to end systematic racism. Public statements were made by many institutions in an effort to publicly recognize the existence of racial inequalities and also show support of the movement. Most professional sports teams in the United States were not exceptions. By the time of writing, more than 94% of the professional sport franchises in the four major leagues in the United States released a BLM-related statement on their communication channels (e.g., website and/or social media). Given that prompt responses were made by most of the professional teams, the current study attempted to explore all publicly-available BLM statements via text analysis techniques.

For the additional context of the current study, following the murder of George Floyd, sport leagues, teams, and athletes have engaged in social activism by supporting the BLM movement. For instance, leagues such as the MLB, NBA, and NHL held moments of silence, and displayed slogans and symbols to oppose racism (5). Between May 27 and June 11, 2020, 118 professional teams (out of 123 teams) from four major professional leagues (i.e., NFL, MLB, NBA, and NHL) released official statements related to BLM. Official statements on BLM can be considered as a proxy of teams' stances on racial injustice issues around the BLM movement. It is important to note that teams have the choice to control their narratives in their statements sent out to the public. For instance, some teams can choose to highlight deeply-rooted racism in society and express anger against police brutality, while others can be more neutral or focus more on bringing the community together. In other words, some teams can choose to focus less on criticizing racial injustice or police brutality.

Although it is up to each team to craft official statements shared to the public, we contend that teams in different leagues might show differences in their narratives and word choices within their statement. It is based on the premise that major sport leagues in the U.S. have shown somewhat different responses and actions toward race issues (3). For instance, the NFL has struggled for years to contend with Colin Kaepernick's activism efforts (6) and their approach in hiring Black coaches (6). On one hand, the MLB's approaches to issues of racial injustice were disparaged for lacking coherence (7), and the NHL was criticized for being sluggish to support anti-racism protests (8). On the other hand, the NBA took the lead on boycotting games in protest against police brutality, which sparked a chain reaction for other leagues and teams to take a pause in their scheduled games (9). Considering such discrepancies in the league-wide reactions and sense of urgency in promoting the BLM movement, we investigated 118 professional team's official BLM statements. Ninety-four percent of the four major leagues' (i.e., NFL, MLB, NBA and NHL) teams issued an official statement promptly after the murder of George Floyd on May 25, 2020, and we analyzed all of those statements by employing text analysis methods (i.e., word cloud and sentiment analysis). Statements were grouped into the league and different regions to account for contextual variation in the word choices and sentiments.

The current study is the first known attempt to investigate professional sport organizations' official statements addressing social activism. Findings of the study advance sport communication literature by offering empirical evidence whether word choices and sentiments around the public statements promoting the BLM movement differ at the league level. Our findings will also shed new insights for scholars and communication strategists to utilize public statements as a proxy of the organizations' stances on specific targeted issues.

### Text analysis on written statements

Text provides important information about messages and sources' positions toward the issue. Researchers in various fields such as political science, business management, communication, and marketing have long been using text analysis to examine written statements (e.g., 10, 11). A variety of research has explored written statements as a form of communication from CEO's statements, company's mission statements, government's official statements, to newsletters sent to stakeholders (e.g., 12–14). Prior research has adopted various textual analysis methods, such as content analysis, word cloud analysis, and sentiment analysis to offer main keywords and positive-negative valence of the statement. For instance, Na et al. (16) used text-mining and balanced scorecard techniques to investigate CEO's messages on websites. The authors extracted significantly repeated keywords from the CEO's message on the organization's website and examined the relationship between the keywords and the organization's financial ratios. The findings highlighted the importance of a website's disclosure and provide useful information for managers in prioritizing strategies and crafting messages on the websites. In the context of annual corporate social responsibility (CSR) reports by firms, Liu et al. (15) utilized text analysis to extract keywords with word frequency. The study also performed linguistic analysis to analyze the corporate characteristics presented in the writing of their CSR report. Findings of the study can inform company's CSR-related decision-making and their stance on such issues.

Some researchers have also utilized interactive visualization tools, such as word cloud analysis. Word clouds are a straightforward and effective visualization tool that offer an intuitive and visually appealing overview of the words that are used most frequently within a given text (e.g., 16). For instance, Kulevicz et al. (17) utilized a word cloud approach to examine corporations' sustainability reports and identified several keywords that appeared in most of the analyzed articles. Those identified keywords represent the corporation's overall stance and involvement in the sustainability report. Researchers have even adopted the word cloud approach as a starting point for deeper analyses (e.g., 18, 19).

### Social activism responses in sport

Sport serves as an important platform for drawing attention to social issues and promoting positive social change (20, 21). It largely drives discussion, debate, and dissent (22). Today, an increasing number of athletes are willing to participate in social activism. Meanwhile, sport organizations have become the target of social movement activity and the sites of social activism that collaborates or responds to social movements (2). Sports organizations cannot remain silent anymore and have to make strategic decisions related to athlete activism (23). Athlete activism can impact leagues or teams' fanbase (e.g., 24), attendance (25–27), and brand image (28, 29). One previous study also found that attitudes to social movements are positively related to attitudes toward the leagues' responses, which affect league credibility (30).

Therefore, how a sport organization defines, frames, and presents athlete activism is crucial (23, 24). When organizations make decisions about supporting particular social issues, they should be careful if their actions are aligned with their corporate social responsibility efforts and organizational values (23, 31). In addition, language and context play an important role in how a sport organization communicate their activism (32, 33). Sports leagues such as the NFL may reframe athlete activism in media communication to protect the brand (28). Thus, it is important to understand how sports leagues develop managerial activism communication and the effectiveness of their communication strategies.

The current literature in athlete activism has largely explored how fans perceive and react to social activism in sport (e.g., 34, 35). A few studies investigated how athlete activists perceive activism and speak up on social issues (e.g., 36–38). Some researchers also explored the effect of different activism types, activism efforts, and audience characteristics (e.g., 34, 39). Further understanding of sport organization participation and responses to social activism is needed due to their critical roles in social activism (2).

In sport, a handful of studies have adopted text analysis methods to explore social activism responses by team owners and athletes. For instance, McGannon and Butryn (40) examined public statements made by 32 NFL team owners/CEOs responding to President Trump's speech on September 22, 2017, where he took aim at NFL players kneeling during the National Anthem, and directing owners to punish those athletes. After Trump's speech targeting NFL owners and athlete activism, all 32 NFL team owners/CEOs publicly responded in defense of players' first amendment free speech rights. Those statements were available on teams' social media (e.g., Twitter) and other news outlets. The authors applied qualitative critical discourse analysis and identified that the statements tend to avoid explicitly addressing institutionalized racial violence and inequalities, yet highlighted abstract values of equality for all. The authors also found that the statements adopted a “functionalist” viewpoint, which called for “unity” and “equality for all through football,” further downplaying the racial politics and injustice to Black communities. The functionalist view is identified by the NFL owners continuing to emphasize football as a vehicle and space through which U.S. neoliberal values of meritocracy are enabled and realized. The authors also identified “post-racial nationalism” in the discourse analysis which highlights symbolic displays of American unity (e.g., honoring the flag, National Anthem) but less explicit mention of race. Such a viewpoint further downplays racial violence and inequalities yet promote color-blind ideology (41). At the same time, sports media and others echoed the claims to unity and dropped the discussion on social justice (42). Demands for unity does not resolve the causes of disruption when communicating to pursue social justice (42). Similarly, Lopez (28) analyzed the NFL management responses to player protests and President Trump's comments including television broadcasts and online statements, and argued that the league reshaped athlete activism to obscure the impacts of athlete activists and protect its media brand. In terms of athletes' responses to social activism, Schmittel and Sanderson (38) analyzed 265 tweets from 125 NFL players that discussed the verdict of George Zimmerman who shot 17-year-old Trayvon Martin. The authors applied constant comparative procedures and investigated how NFL players talked about the case. Contrary to the NFL's responses to President Trump's speech and social activism, the NFL players explicitly criticized the American Justice System and linked the case to larger social and racial issues in the United States.

In the wake of the widespread BLM movement after the murder of George Floyd, some scholars have also explored how the league and fans responded to such racial advocacy movements (e.g., Brown-Devlin, (44), Seaton et al., (45)). Previous studies have found that league responses can be influenced by internal stakeholders and external interests. Brown-Devlin (2023) investigated athletes and NFL employees using their own social media as a platform to demand more organizational actions and statements from the commissioner. The study found that collective voicing from NFL athletes and employees prompted an initial and small movement toward more advocacy at the organizational level. This study highlights how voices from internal stakeholders using social media can influence organizational decision-making. Williams (43) also found that during 2020, NBA and WNBA players pushed the leagues to engage in the BLM movement and promote social justice. Donahue (33) explored how the NFL responded to the Colin Kaepernick protests in 2016–2017 and how the league responded to protests during the BLM movement in 2020. The author analyzed protest responses from NFL personnel including the commissioner, team owners, and NFL executives. During 2016 and 2017, the NFL personnel condemned Kaepernick for his actions and centered on national pride and the National Anthem. However, during the BLM movement in 2020, many of these NFL personnel called for unity and understanding for racial unity. The NFL responses changed drastically from Kaepernick's protests to the BLM movement. The author suggests that the league's responses to social movement were influenced by external interests including NFL fans and the public.

On the other hand, Seaton et al. (2022) explored fan reactions to the NFL teams' racial advocacy and promotions (e.g., helmet stickers, name displays) that took place at the beginning of the 2020 season. Twitter replies were analyzed and the results showed fans' resistance toward racial advocacy as a function of fan identity (in-group) and political orientation (conservative). Gill (44) and Sanderson et al. (24) examined how fans responded to St. Louis Rams players' “hands up don' shoot” protest on field on social media and discovered divisive opinions on the athlete activism. Although a handful of research has examined various communication sources related to racial justice advocacy in the NFL, little research has examined official statements from professional teams across the four major sport leagues (i.e., MLB, NBA, NFL, and NHL). Thus, do teams in different leagues show any differences in addressing racial injustice and police brutality in their statements? For instance, what are the dominant words used in the statements across leagues? Which league(s) demonstrate more critical (or positive) tones towards the racial justice issue? We aim to address these questions by investigating sport teams' official statements on the BLM movement, and intend to offer empirical insights on whether collective responses across the major professional sport leagues show any varying characteristics.

### Purpose and research questions

In the current study, we sought to provide initial evidence of how teams from different leagues responded to the BLM movement and how team statements differed across each sports league and geographic region. To our knowledge, our study is the first to explore official BLM statements made by professional sport teams. In doing so, we hope our study will reveal interesting patterns on whether there are any differences in crafting messages to their respective audiences. Findings from our study will lend communication insights for league and team public relations specialists about message development and its structure. In particular, we propose two research questions:

**RQ_1_:** What are the most frequently used words across all BLM statements, and how are words used differently across the leagues?
**RQ_2_:** Are there any differences across the “Big Four” North American professional sports leagues and geographic regions on the statements' word count and overall sentiment?

## Method

### Data collection

We retrieved the content of BLM statements from four men's professional sports teams (i.e., MLB, NBA, NFL, and NHL) that made related posts on Twitter shortly after the murder of George Floyd from the period of May 27 to June 11, 2020. When applicable, word counts from the BLM statements were derived as some were posted as images. In all, we collected statements from 118 teams among the 123 franchises across the four leagues. During the sample period, five teams did not post an official statement on Twitter. Twitter was selected as a platform as sport organizations often use it as an efficient communication tool to handle crisis situations and it is also considered more interactive and a faster instrument for relationship building with the target audience (e.g., 45–47). In fact, all of those teams released a statement using their teams’ official Twitter account. For additional comparisons, teams located in the United States were segmented by geographic region based on the United States Census Bureau (48) map of Regions and Division (West, Midwest, South, and Northeast). This addition of regional breakdown is based on the literature that accounts for variations in racial and ethnic equity improvement across Census Regions (e.g., 49, 50). Given the small number of teams located in Canada, they were classified at the country level.

### Data analysis

Analyses were performed in Python (Version 3.8.8) and RStudio (Version 1.3.1056) for Mac. Jupyter Notebook was used to write code and install all the necessary packages and libraries for our analyses in Python. We used the *nltk* package in Python to preprocess data for further analysis. Preprocessing data is a key step for any Natural Language Processing and has a significant influence on the results (51). Without preprocessing, the frequent words found in the statements could be the ones commonly used in English language (e.g., “the”, “and”, “to”). Meanwhile, some highly used words could not be identified since they are in different grammatical tenses. We followed and modified the procedure suggested by Allahyari et al. (52) for text analysis to remove noise from the dataset: (1) converted uppercase to lowercase, (2) removed punctuations marks and symbols, (3) tokenized the statements into words, (4) removed stop words, and (5) lemmatized words. Since the name “George Floyd” was frequently mentioned in the statements, we customized it as one word. To explore the word frequency in all BLM statements and the differences of words used across four leagues (RQ1), we first generated word clouds by using the *wordcloud* package in Python. Word clouds are widely used in text mining (e.g., 53, 54), and the technique has been identified as an effective approach to visualize text and explore words with the highest frequency (16). Word clouds can also serve as a preliminary step for text analysis (19). To further investigate the words used by the four leagues, we generated term frequency and document frequency tables of the highly used words in the statements. Term frequency measures how many times a word is present in a document, while document frequency is the number of documents containing that word (55). Term frequency and document frequency are both common in text categorization to discover significant words and examine the content of corpora (56). In this study, we first treated statements of each league as a document and used the *pandas* package in Python to calculate term frequency. To avoid the bias that a team may use a word multiple times in their statement, we then treated each statement as a document and statements of each league as corpus to measure document frequency. A series of follow-up chi-squared tests were performed with the *scipy* package in Python to determine if any words were used significantly differently among four leagues. Baron et al. (56) suggest that the chi-squared test can be applied on a 2 × 2 table to compare frequencies of words or other linguistic features across different corpora.

For statistical analysis of the BLM statements (RQ2), we conducted a series of generalized linear regression models with Holm-Bonferroni *post-hoc* tests. Specifically, we examined the individual impact of professional sports league and geographic region on the raw word counts of BLM statements released by each team. To account for the overdispersion in word counts, a quasi-Poisson distribution was specified. A follow-up sentiment analysis was also performed to determine the proportion of positive and negative words utilized in each team's statement. Words were categorized using the binary dictionary created by Hu and Liu (61) through the *tidytext* package in R. Similar generalized regression models that assumed linearity were utilized to determine the differences among leagues and regions.

## Results

### RQ1: usage of words across BLM statements

**Figure 1:**
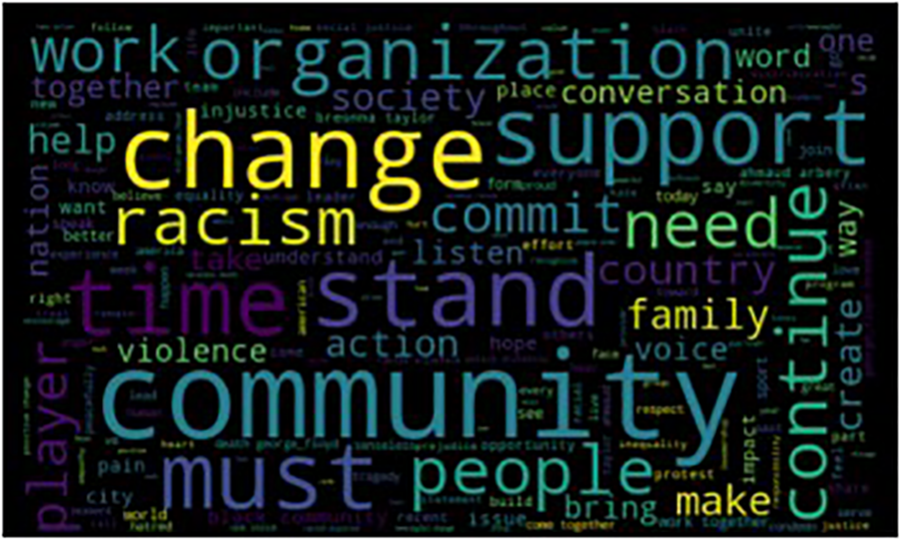
Word cloud visualization of all 118 BLM statements.

Figure 1 shows the word cloud of all BLM statements. Overall, “community” was the most used word across all the statements, followed by “change”, “racism”, “stand”, and “support”. Similar patterns existed in the statements of each league in that “community” and “change” were still highly used. Interestingly, “racism” was listed as a frequently used word in the statements of the MLB, NBA, and NHL teams. However, it did not appear to be frequently used in statements by NFL teams (listed as 12th frequently used word) compared to other leagues.

Table 1 shows the term frequency of the top-20 words used in the BLM statements across all leagues. “Community”, “racism”, “change”, “support”, and “time” were the top-5 words used in all leagues. The results indicated that the statements focused on condemning racism, bringing people together, and expecting positive outcomes of the community. Different patterns were also shown across the leagues. Although “racism” was one of the top-5 most frequently used words by MLB, NBA, and NHL teams in their statements, it only ranked 12th among teams in the NFL. Some action words like “support”, “listen”, “action”, “help”, and “conversation” were frequently used in statements by NFL teams, which appeared to be relatively less common in the statements from teams in other leagues. At the same time, the NHL seemed to use more powerful words to criticize the social issues, including “racism”, “violence”, “injustice”, “condemn”, “bigotry” and “protest”. After exploring some of the different patterns across the leagues, we used document frequency to measure how many statements contained the following words: “racism”, “support”, “violence”, “protest”, “bigotry”, “condemn”, “respect”, “conversation”, “action”, “help”, “listen”, “commit” and “society”. The goal was to further investigate different words used across the leagues.

**Table 1:** Term frequency of top 20 words used across all leagues.

MLB	*n*	NBA	*n*	NHL	*n*	NFL	*n*
Community	38	Change	48	Racism	35	Change	49
Racism	35	Community	43	Community	34	Community	47
Stand	27	Time	34	Together	23	Player	42
Change	25	Racism	31	Stand	19	Work	37
Black	18	Work	29	Violence	19	People	32
Together	18	Black	27	Work	16	Support	31
Commit	17	Organization	27	Change	16	Listen	29
Organization	17	Must	26	Need	15	Continue	28
Injustice	16	Together	25	Society	14	Must	26
Work	16	George_floyd	24	Support	13	Make	25
Live	15	Stand	22	Commit	12	Together	23
Racial	14	Take	22	Injustice	11	Country	22
Society	14	Continue	21	Continue	9	Racism	22
Country	13	People	20	Place	9	Family	22
George_floyd	13	Action	20	Sport	9	George_floyd	21
People	13	Make	20	Condemn	9	Help	21
Continue	12	Live	18	Time	9	Action	20
Support	12	Need	18	Bigotry	9	Conversation	20
Family	12	Use	18	Protest	9	Injustice	20
Time	11	Support	17	Respect	8	Time	20
Total Word Counts	2,011		2,947		1,246		3,477

Table 2 provides the document frequency of the words that were identified to be used differently in the BLM statements across all leagues. “Racism”, “violence”, “bigotry”, and “condemn” appeared in fewer statements from NFL teams compared to teams in the MLB, NBA, and NHL. “Conversation”, “action”, “help”, and “listen” were used in more statements from NFL teams than those from teams in other leagues. The follow-up chi-squared test revealed that the word “racism” was used in significantly fewer statements by NFL teams than teams in the other leagues, *χ*^2^(1) = 8.91, *p* = .002. It also appeared significantly less often in the statements by NFL teams than those from the other leagues, *χ*^2^(1) = 16.81, *p* < .001. The words, “violence”, *χ*^2^(1) = 4.87, *p* = .003, “bigotry”, *χ*^2^(1) = 7.51, *p* = .006, and “condemn”, *χ*^2^(1) = 4.43, *p* = .003, were also used in significantly fewer statements released by NFL teams. However, “conversation”, *χ*^2^(1) = 6.12, *p* = .01, “action”, *χ*^2^(1) = 4.45, *p *= .03, “help”, *χ*^2^(1) = 6.34, *p* = .01, and “listen”, *χ*^2^(1) = 17.50, *p* < .001, appeared significantly more often in statements by NFL teams.

**Table 2:** Document frequency across four leagues.

Word	Professional sport leagues			
	MLB	NBA	NHL	NFL
Injustice	15	12	10	15
Racism	25	23	27	16
Violence	10	12	19	6
Protest	9	5	8	10
Bigotry	4	5	9	0
Condemn	7	9	9	2
Respect	6	8	8	3
Support	9	9	12	15
Conversation	3	5	2	10
Action	8	12	5	15
Help	8	4	5	13
Listen	6	3	2	15
Commit	14	13	10	15
Society	10	7	12	6
Total number of statements	30	29	30	29

### RQ2: word count and geographical differences among BLM statements

The loglikelihood ratio tests revealed a significant effect of both league [*χ*^2^ (3) = 31.26, *p* < .001] and region [*χ*^2^ (4) = 22.79, *p* < .001] on BLM statement word counts. Specifically, NHL teams [estimated marginal mean (*EMM*) = 81.18, standard error (*SE*) = 13.61] had shorter statements than NBA (*EMM* = 179.24, *SE* = 22.58; *OR* = 0.45, *p* < .001) and NFL teams (*EMM* = 221.95, *SE* = 26.53; *OR* = 0.37, *p* < .001). In addition, MLB teams (*EMM* = 128.37, *SE* = 18.39) had shorter BLM statements than NFL teams (*OR* = 0.58, *p* = .004). Teams located in the South (*EMM* = 223.75, *SE* = 23.07) had longer statements than those located in the Midwest (*EMM* = 140.23, *SE* = 18.25; *OR* = 1.59, *p* = .03) and Western (*EMM* = 102.09, *SE* = 15.57; *OR* = 2.19, *p* < .001) regions of the United States.

**Figure 2:**
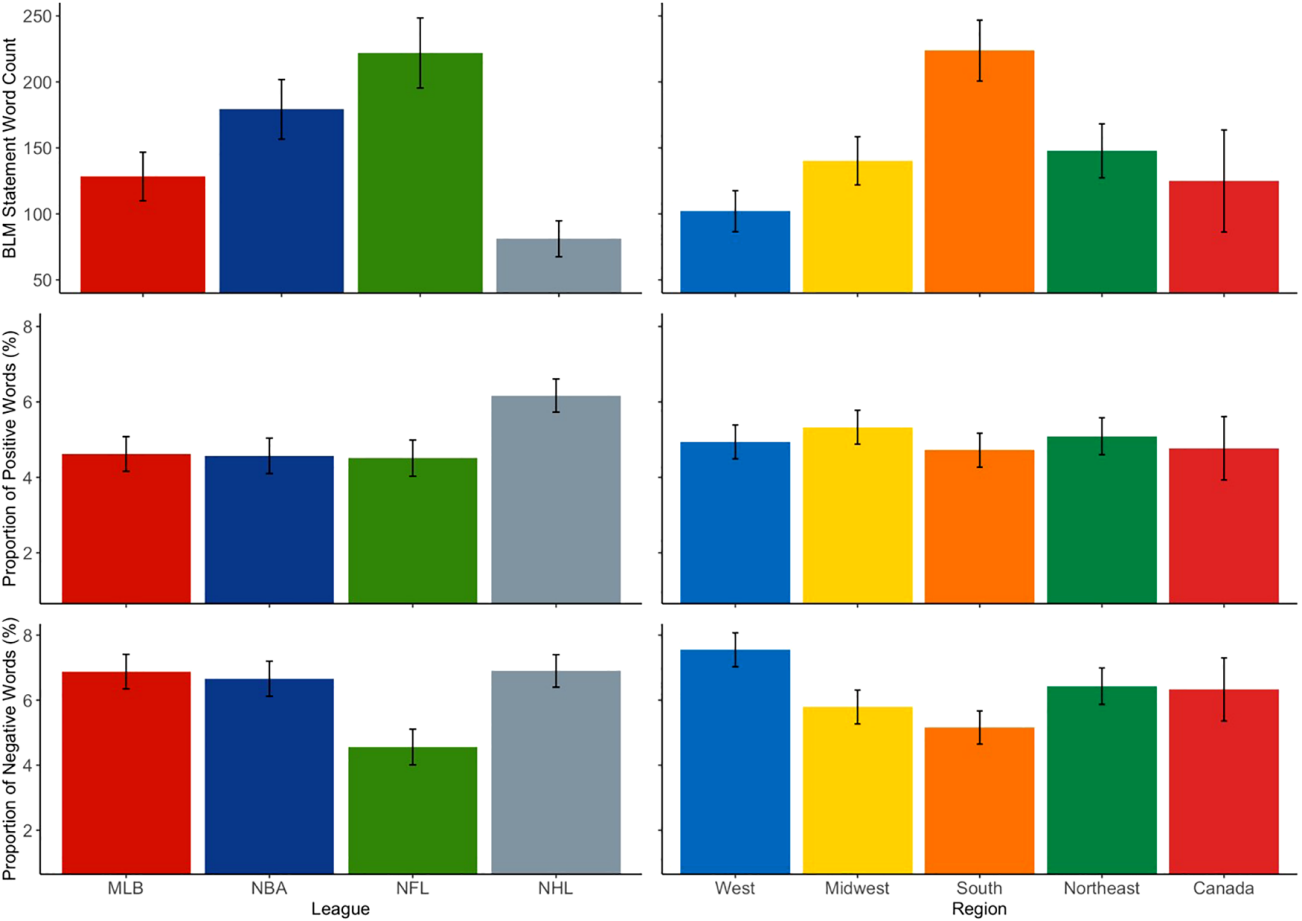
Estimated marginal means plots for the effect of league and region on BLM statement word counts and sentiment proportions. Error bars denote standard error.

Although the loglikelihood ratio test revealed an omnibus effect of league on the proportion of positive words utilized in BLM statements [*χ*^2^ (3) = 8.85, *p* = .03], there were only marginal differences revealed by the *post-hoc* comparisons. Herein, statements released by NHL teams contained a greater proportion of positive words (*EMM* = 6.17%, *SE *= 0.44%) compared to those by MLB (*EMM* = 4.62%, *SE* = 0.46%; *p* = .07), NBA (*EMM* = 4.57%, *SE* = 0.47%; *p* = .07), and NFL teams (*EMM* = 4.51%, *SE* = 0.48%; *p* = .07). However, there were significant overall effects of both league [*χ*^2^ (3) = 14.29, *p* = .003] and region [*χ*^2^ (4) = 11.62, *p* = .02] detected for the proportion of negative words contained across statements. Specifically, MLB (*EMM* = 6.88%, *SE* = 0.53%; *p* = .007), NBA (*EMM* = 6.66%, *SE* = 0.54%; *p* = .02), and NHL teams (*EMM* = 6.90%, *SE* = 0.50%; *p* = .01) used a higher proportion of negative words than teams in the NFL (*EMM* = 4.56%, *SE* = 0.55%). Across regions in the United States, teams located in the South (*EMM* = 5.16%, *SE* = 0.51%) used a smaller proportion of negative words in their statements than those in the West (*EMM* = 7.55%, *SE* = 0.52%; *p* = .01). A visual summary of the results is presented in Figure 2.

## Discussion

The present study sought to investigate American men's professional sport teams' official BLM statements issued following the murder of George Floyd. Such official statements can serve as the organization's stance on systematic racism and violence against the Black community. Applying text analysis methods, empirical findings revealed that the four professional sport leagues seemed to have different strategies in crafting their BLM statements. There were also several variations by geographic region. For instance, sports teams located in the South had the longest statements and used fewer negative words in their statements. Overall, NHL teams had the shortest statement length, while NFL teams had the longest statements on average. The Southern United States has had a long history of discrimination against Black and other minority groups (57), and the use of longer statements could be an implicit way to compensate for this checkered past. Similarly, the NFL has been criticized for the suppression of Black agency, consistently weak responses to social justice efforts (28), and racist practices in the selection and evaluation of talent and leadership (58, 59). Thus, the increased frequency of words may also be a way to publicly showcase the league's endeavor to support the BLM movement.

Regarding the actual statement content, NFL teams used the word “racism” the least in their statements. In addition, only a few statements released by NFL teams contained the word “violence”, “bigotry”, and “condemn”, which were mentioned quite often in the statements by teams from the MLB, NBA and NHL. It also appeared that NFL teams avoided using many negative words in their statements, given the relatively low proportion of such words as revealed by our sentiment analyses. In particular, “racism” was used in significantly fewer statements by NFL teams than those in the other leagues. This avoidance of using negative words could stem from perceptual linguistic biases that people hold. That is, negative words tend to be perceived with greater potency and could lead to more negative evaluations than when positive messages are presented (60). These findings also show empirical support for the critical discourse analysis (40) that examined the NFL owners' responses to President Trump's speech about athlete protests. In their analysis, the authors identified two emerging discourses in the NFL owners' responses: post-racial nationalism and functionalism. Post-racial nationalism discourse prioritized traditional American values and notions of inclusive citizenship and ideals of equality for “all”, which displaced explicit mention of race and police violence. Our quantitative analyses also show evidence that the NFL teams used the word “racism” significantly less than other leagues' teams. The word appeared 12th most frequently used among NFL teams, while it appeared as the 1st (NHL), 2nd (MLB), and 4th (NBA) in other leagues. We contend that post-racial nationalism discourse documented in the NFL owners' previous statements is also reflected in the NFL teams' BLM statements that they are devoid of institutionalized racial violence and inequalities (61).

Functionalism discourse noted in McGannon and Butryn's (40) critical analysis is also further evidenced in our empirical findings. The finding that the NFL teams used significantly more positive words such as “conversation”, “action”, “help”, and “listen” than other leagues portrays how the NFL teams aim to highlight football as a vehicle and space to bring people together. As such, we contend that the functionalist rhetoric adopted by the NFL owners is also further reflected in our findings that the most frequently used words in the documents tend to be positive (e.g., “community”, “change”, “people”, “support”, “listen”, “action”, etc.). That is, rather than criticizing the racial issues, the NFL teams focused more on proposing initiatives to address the problems (e.g., building on conversations, listening to different voices, helping people in need, and taking actions to change the current situations) without directly naming or condemning police brutality that instigated the BLM movement. In a corporate context, research by Korschun et al. (62) showed that the avoidance of political stands can introduce a level of risk for some companies. Thus, the NFL may be intentionally using the statements to match their words to actions in a sense. Supporting research by Zhou and Dong (63) has illustrated the importance of coordinating stances with actions, finding that corporations can avoid negative responses from consumers even if they partially realize the goals they publicly put forward. For instance, after the fallout from the protests over the murder of George Floyd, the NFL was one of the first major sports leagues to announce that they would commit $250 million over the course of 10 years “to combat systemic racism and support the battle against injustices faced by African Americans” (64). In addition, after publicizing its financial commitment, the NFL allowed players to choose one of six messages to place a decal on the back of their helmets: “End Racism,” “Stop Hate,” “It Takes All of Us,” “Black Lives Matter,” “Inspire Change,” and “Say Their Stories.” These noticeable changes (allowing a helmet decal) and financial commitments further demonstrate the league's different response to the similar issue from 2016 to 2017 as documented by Donahue (33). Although many are skeptical of the league's authenticity in these efforts to tackle systemic racism including various players (65), the NFL may be aligning the statements with their efforts to enhance public perception.

Although not reported in the main analysis, there was only one NFL team that mentioned Colin Kaepernick, who protested in 2016 to raise awareness of police brutality toward Black communities. It is interesting to see how the NFL teams refrained from the acknowledgement of police brutality and social injustice in the Black community yet positioned themselves to call for unity and positive change (cf. 40). Thus, our findings advance sport activism research by demonstrating how institutionalized communications differ across sport leagues.

In contrast, the NBA, MLB, and NHL teams seemed to focus more on condemning racism and emphasizing the severity of the social issues. The NBA, as well as the WNBA, have been lauded for their work in addressing systemic racism (66). Thus, it is not surprising that the NBA had a strong response to the BLM movement through their statements, given that they focused on criticizing the injustices that transpire in society. In the past, the MLB has fluctuated in its responses to social activism, with periods of positive social activism dating back to the days of Jackie Robinson to a seemingly silent period lasting for decades. However, with the resurgence of the BLM movement in 2020, the league had a much stronger stance on combating systemic racism (67). Players such as Aaron Judge, Andrew McCutchen, and Mookie Betts were featured in a video put forth by the league to unify the community and seek out change. Consequently, the negative tone of MLB teams' statements may be perceived as a welcome shift from previous practices where athletes were discouraged from speaking out.

Our findings also showed that teams in the NHL used both positive and negative words most often. We find this to be a case where the league was accused by many players, commentators, and fans for a lack of solidarity with BLM protests (8, 68). We argue that increased criticisms due to their lack of urgency in responding to the call for institutional action might have influenced teams to take a more condemning tone towards racism yet calling for positive change. However, it should also be noted that the NHL teams' BLM statements were the shortest compared to all other leagues. Perhaps shorter statement lengths might speak to the NHL being the least racially diverse sport league in North America, with less than 5% of players being Black or people of color (69). After issuing those statements, the NHL also initiated anti-racism efforts by requiring all employees to participate in an inclusion learning experience that focuses on anti-racism, unconscious bias, micro-aggressions and cultural competency (70). The NHL also showed an effort to make cultural change within the league by creating the Executive Inclusion Council, which will be committed to promote more inclusive thinking and opportunities for positive change (70). Although their BLM statements' length was shorter relative to other leagues, the league announced that teams will adopt additional measures to fight against racism and make the league more welcoming and inclusive.

### Limitations and directions for future research

The present study has several limitations. First, while word cloud analysis is used as a visual comparative tool across four professional sport leagues, it does not necessarily signal importance of those words (71). It shows how specific words were frequently used, but it does not indicate how the audience assigned meanings or importance to those words. Although we used a more quantitative approach to analyze the statements, future research can complement this study by applying critical discourse analysis (cf. 40) to further explicate sport organizations' motivation for and underlying meanings of public written statements. Discourse analysis would help researchers to uncover the motivation and underlying meanings behind those statements that the quantitative approach (e.g., word cloud analysis) might not able to examine. Therefore, the combination of both quantitative and qualitative textual analyses will offer much deeper insights into the written statements by professional sport teams. Second, while the focus of the study was on men's professional leagues, we acknowledge that including women's sports league such as the WNBA might exhibit different patterns from men's sports leagues. It should be noted that the WNBA teams and athletes have long been leaders in advocacy on social justice and equity (72) and therefore, future research should incorporate WNBA teams' statements in those situations. Such comparative analysis would offer additional insights on how men's and women's league might differ on crafting those official statements. In addition, future research could expand to consider athletes speaking out on social justice matters and how it might differ from communications at the organization level. Third, the current study did not incorporate sport fan reactions. It remains unknown whether the overall sentiment or word choices had any influence on audience engagement. Future studies can examine how various message features (e.g., length, word choices, sentiment) can influence the audience's reaction, such as liking or sharing messages. When assessing audience reactions, researchers should consider fan attachment and loyalty (73) as they might influence how written statements related to social justice movemnts are perceived. Further, audience characteristics (e.g., demographics, political stance) can be incorporated to see whether they influence perceptions toward the team's BLM statement. It is also important to consider other social media platforms as the audience demographics and psychographics might differ based on the platform. Audience engagement studies will further advance our understanding of the persuasive effectiveness of institutionalized statements related to sociopolitical issues.

## Data Availability

The raw data supporting the conclusions of this article will be made available by the authors, without undue reservation.
